# Separation of Heat-Stable Antifungal Factor From *Lysobacter enzymogenes* Fermentation Broth via Photodegradation and Macroporous Resin Adsorption

**DOI:** 10.3389/fmicb.2021.663065

**Published:** 2021-05-13

**Authors:** Bao Tang, Lingtian Wu, Jinzi Wang, Weibo Sun, Yancun Zhao, Fengquan Liu

**Affiliations:** ^1^Jiangsu Key Laboratory for Food Quality and Safety-State Key Laboratory Cultivation Base of Ministry of Science and Technology, Institute of Plant Protection, Jiangsu Academy of Agricultural Sciences, Nanjing, China; ^2^School of Chemistry and Chemical Engineering, Jiangsu University, Zhengjiang, China; ^3^College of Biological and Food Engineering, Changshu Institute of Technology, Changshu, China; ^4^College of Plant Protection, Nanjing Agricultural University, Nanjing, China; ^5^School of Life Sciences, Jiangsu University, Zhengjiang, China

**Keywords:** *Lysobacter enzymogenes*, photodegradation, macroporous resin adsorption, NKA resin, heat-stable antifungal factor

## Abstract

Heat-stable antifungal factor (HSAF) is produced by the fermentation of *Lysobacter enzymogenes*, which is known for its broad-spectrum antifungal activity and novel mode of action. However, studies on the separation of HSAF have rarely been reported. Herein, alteramide B (the main byproduct) was removed firstly from the fermentation broth by photodegradation to improve the purity of HSAF. Then, the separation of HSAF via adsorption by macroporous adsorption resins (MARs) was evaluated and NKA resin showed highest static adsorption and desorption performances. After optimizing the static and dynamic adsorption characteristics, the content of HSAF in the purified product increased from 8.67 ± 0.32% (ethyl acetate extraction) to 31.07 ± 1.12% by 3.58-fold. These results suggest that the developed strategy via photodegradation and macroporous resin adsorption is an effective process for the separation of HSAF, and it is also a promising method for the large-scale preparation of HSAF for agricultural applications.

## Introduction

The Gram-negative *Lysobacter* bacteria, belonging to the *Xanthomonadaceae* family of Gammaproteobacteria, are ubiquitous soil and freshwater environmental microorganisms. Members of this genus have been regarded as promising biological control agents against crop fungal and bacterial diseases because of their characteristics of fast growth, easy maintenance, and genetic amenability to bioengineering (Chen et al., 2018). A typical representative and well-studied species is *Lysobacter enzymogenes*, which exhibits antimicrobial potential by the production of not only lytic enzymes, but also a variety of promising antibiotics, especially an antifungal antibiotic called Heat-Stable Antifungal Factor (HSAF) (Zhao et al., 2017).

HSAF is a polycyclic tetramatemacrolactam (PoTeM), and its chemical structure contains a unique macrolide system, a tetralic acid structural unit and a 5,5,6-tricyclic skeleton; its structure is distinct from those of other existing antifungal drugs or fungicides (Yu et al., 2007; Lou et al., 2011). Moreover, HSAF exhibits a highly potent antagonistic activity against bacteria, unicellular algae, and nematodes, especially fungal species. More importantly, its mode of action on pathogenic fungi is novel and different from those of the previously reported commercial fungicides. Previous studies have indicated that HSAF inhibits the polarized growth of filamentous fungi by disrupting the biosynthesis of sphingolipids, which differs between fungal and mammalian cells (Li et al., 2006). Regarding its distinct structure and novel mode of action, HSAF has great potential for being used as biological pesticides for green and safe agricultural production. At present, it is an attractive subject, and much progress has been made in investigating its biosynthesis mechanism, identifying regulatory factors, and improving its yield. For example, the key genes and biosynthesis pathway of HSAF have been clearly studied (Yu et al., 2007; Lou et al., 2011; Li et al., 2014, 2018). Several key factors involving the regulation of HSAF have been identified into two categories: positive [*Le*DSF (Li et al., 2020), Clp (Wang et al., 2014), and Lsp (Wang R. et al., 2017)] and negative regulatory factors [LesR (Xu et al., 2017), PilR (Chen et al., 2017), and LetR (Wang P. et al., 2017)]. In addition, the production of HSAF has been greatly improved to 440.26 ± 16.14 mg/L by optimizing the fermentation medium and conditions (Tang et al., 2018a, b). However, studies on the separation and purification of HSAF from the fermentation broth have rarely been reported.

The conventional method for separating HSAF from the fermentation broth is liquid-liquid extraction followed by column chromatography or preparative high-performance liquid chromatography (HPLC) (Tang et al., 2018a). However, the whole purification process is time-consuming and laborious and is characterized by excessive solvent wastage, lengthy operation techniques, poisonous residual solvents (e.g., ethyl acetate), and low product recovery, making the method unsuitable for large-scale industrial production. Furthermore, this separation method results in some other PoTeM compounds, especially alteramide B (ATB) with the high concentration of 258.81 mg/L, coexisting with HSAF because of their similar structures and chemical properties (Tang et al., 2019), leading to a low purity of the final HSAF. Therefore, it is urgent to develop an effective method for the preparation of HSAF from *L. enzymogenes* fermentation.

Macroporous adsorption resins (MARs), a type of highly cross-linked and non-ionic chromatographic materials, can selectively adsorb targeted constituents from aqueous and non-aqueous systems through electrostatic force, hydrogen bonding interaction, complexation and size sieving action (Chen and Zhang, 2014).

With the advantages of excellent selectivity and high recovery and efficiency, the MAR-based separation method has been widely employed for separating and purifying target compounds from many natural products, such as flavonoids (Dong et al., 2015; Huang et al., 2017), alkaloids (Cao et al., 2018), and polyphenols (Xi et al., 2015). Moreover, previous studies have reported the adsorption of many compounds in fermentation broths by MARs, such as in the separation and purification L-methionine from *E. coli* fermentation broth by D72 resin (Xiong et al., 2019) and the large-scale preparation of high-purity menaquinone-7 from *Bacillus subtilis* natto fermentation medium by HPD722 resin (Fang et al., 2019); this proves the feasibility and provides support for our research. More importantly, owing to MARs possessing some special characteristics such as high mechanical strength, good acid and alkali resistance, simple operation, low cost, easy regeneration, and scale-up capability, the MARs method is more suitable for large-scale separation processes and thus, have gained increasing interest in industrial practices (Li et al., 2012; Liu B. Y. et al., 2016; Leyton et al., 2017). Therefore, it is feasible to use MARs to separate and purify HSAF from fermentation broths.

The objective of this work was to develop an effective method for preparing HSAF from the fermentation broth of *L. enzymogenes*. First, a photodegradation reaction was carried out to prevent the interference of ATB. Then, 14 MARs with different physical and chemical properties were investigated to select the optimal resin by evaluating their static adsorption and desorption properties for HSAF. Meanwhile, the adsorption kinetics and isotherms of HSAF on the selected resin were studied to improve the sorption process and predict the resin performances. Finally, the dynamic adsorption and desorption processes were systematically optimized for the purification of HSAF.

## Materials and Methods

### Pretreatment of MARs

14MARs of net grade were purchased from Tianjin Haoju Resin Technology Co., LTD. (Tianjin, China), and their physical and chemical properties are summarized in Supplementary Table 1. Prior to use, these resins were treated according to the manufacturers’ recommendation. The resins were soaked in ethanol and shaken overnight. Afterward, they were washed with deionized water until there was no smell of ethanol and were then stored at 4°C.

### Preparation of Fermentation Broth

The HSAF fermentation broth was produced by *L. enzymogenes*OH11, reported in a previous study (Tang et al., 2018a). As pretreatment, the crude fermentation broth was filtered by six layers of gauze to remove some soybean powder residues; the concentration of HSAF in the broth was about 300 mg/L.

### Illumination Experiment

The filtrated fermentation broth was loaded into a 1,000 mL serum bottle, and incubated in an illuminating incubator (Guangdong Medical Device Factory, Shaoguang, Guangdong) under 18W fluorescent light for 2 days. The samples were withdrawn at given time intervals (every other day) to analyze the photodegradation degrees of HSAF and ATB by HPLC (Shimadzu LC-6AD, Japan).

### Static Adsorption and Desorption Experiments

To acquire the most suitable macroporous resin to separate HSAF from the fermentation broth, the adsorption and desorption properties of the different MARs were evaluated using the following methods.

Static adsorption tests: Pre-weighed amounts of hydrated resin (equal to 0.62 g of dry resin) and 50 mL of HSAF fermentation broth were added into a 250-mL conical flask with a stopper and then shaken at 180 rpm at 32°C for 12 h. After adsorption equilibrium was reached, the resin was removed from the sample solution by filtration with three layers of gauze. The filter liquor was extracted, and the HSAF concentration was analyzed by HPLC.

Static desorption tests: The filtered resin was washed twice by deionized water, and then desorbed with 50 mL of ethanol. The conical flask was continually shaken under the same conditions as in the adsorption process, and the desorption solution was directly analyzed by HPLC.

The adsorption and desorption capacities and adsorption and desorption ratios were used to evaluate the performance of each resin and calculated according to the following equations:

Adsorption capacity:

(1) $$q_{e}=\left(C_{0}-C_{e}\right)/W\times V.$$

Adsorption ratio (%):

(2) $$E\left(\%\right)=\left(C_{0}-C_{e}\right)/C_{0}\times 100\%.$$

Desorption capacity:

(3) $$q_{d}=C_{d}\times V_{d}/W.$$

Desorption ratio (%):

(4) $$D\left(\%\right)=C_{d}\times V_{d}/\left[\left(C_{0}-C_{e}\right)\times V\right]\times 100\%,$$

where *q*_*e*_ and *q*_*d*_ are the adsorption capacity at adsorption equilibrium and the desorption capacity, respectively (mg/g dry resin); *C*_0_, *C*_*e*_, and *C*_*d*_ are the initial, equilibrium, and desorption concentrations of HSAF (mg/L) in the solutions, respectively; *V* and *V*_*d*_ represent the volume of the initial fermentation broth and desorption solution (mL), respectively; W is the weight of dry resin (g); *E* is the adsorption ratio (%); and *D* is the desorption ratio (%).

The adsorption and desorption kinetics curves of HSAF on the selected NKA resin were studied according to the above method by withdrawing the sample solution at a given interval. The most widely used adsorption models, pseudo-first-and pseudo-second-order models, were used to fit the adsorption kinetic data (Wang et al., 2013). The equations are shown as follows:

Pseudo-first-order model:

(5) $$\ln\left(q_{e}-q_{t}\right)=\ln q_{e}-K_{1}t.$$

Pseudo-second-order model:

(6) $$t/q_{t}=1/K_{2}q_{e}^{2}+t/q_{e}$$

where *q*_t_ is the HSAF adsorption capacity at time t (mg/g dry resin) and can be calculated by Eq. (1), and *K*_1_ (/min) and *K*_2_ (g/mg min) are the rate constants of the pseudo-first-order model and the pseudo-second-order model, respectively.

### Adsorption Isotherms

To evaluate the effect of temperature on HSAF adsorption, the adsorption is other study was performed as follows: The pre-treated NKA resin (equal to 0.62 g of dry resin) was contacted with 50 mL of HSAF fermentation broth at various initial concentrations (*C*_0_ = 50, 100, 150, 200, 250, and 300 mg/L), and shaken (180 rpm) for 12 h at three different temperatures of 27, 32, and 37°C, respectively. Then the equilibrium concentrations of HSAF at different temperatures were determined by HPLC.

Two popular theoretical isotherm models, Langmuir and Freundlich models, were applied to describe adsorption correlations between the adsorbate and adsorbent. The Langmuir equation can be used to describe monolayer adsorption, whereas the Freundlich equation can be used to describe monolayer adsorption and multilayer adsorption (Ares et al., 2012; Wang et al., 2016).

Langmuir model:

(7) $$C_{e}/q_{e}=C_{e}/q_{m}+1/K_{L}q_{m}.$$

Freundlich model:

(8) $$\ln q_{e}=\ln K_{F}+1/n\ln C_{e},$$

where *q*_*e*_ and *C*_*e*_ are the same as mentioned above; *K*_*L*_ (mg/mL) and *K*_*F*_ (mg/mL) represent the Langmuir and Freundlich constants, respectively, *q*_*m*_ (mg/g dry resin) is the theoretically calculated maximum adsorption capacity; and 1/n reflects an empirical constant related to the magnitude of the adsorption driving force.

### Dynamic Adsorption and Desorption Experiments

Dynamic adsorption and desorption experiments were carried out in a glass column (10 × 300 mm) wet-packed with the selected NKA resin (equal to 1.55 g of dry resin). The height of the resin bed was 9.2 cm, and the bed volume (BV) of the packed NKA resin was 7.2 mL. The fermentation broth or ethanol solution was flowed continuously through the glass column at the prescribed flow rate by a peristaltic pump equipped on the 5-L fermentation system (BailunBio, Shanghai, China), and the effluents or desorbed solutions were collected and examined by HPLC.

In the adsorption stage, the effects of the feed flow rate and feed volume on the adsorption capacity of the NKA resin were systematically investigated. When adsorption equilibrium was reached, the adsorbate-laden column was rinsed with 2 BV deionized water and then desorbed with ethanol solution. Several elution solvent systems with different ethanol concentrations, eluent volume, and flow rate for the desorption process were also studied.

### Determination of HSAF and ATB Concentration or Content

The concentrations of HSAF and ATB in the samples were determined by the previously described method but with some modifications (Tang et al., 2018a, 2019). First, 3 mL of fermentation samples were withdrawn and adjusted to pH 2.5 by HCl. Then 0.3 g CaCl_2_ was added to eliminate the emulsification problem in the extraction process, and an equal volume of ethyl acetate was added to extract the target product. The extraction reaction was performed on a vortex at 2,000 rpm for 1 min. After centrifugation at 10,000 rpm for 3 min, 1 mL of the solvent layer containing the HSAF and ATB was separated and ventilated to dryness in a fume hood. Subsequently, the residue was redissolved in 1 mL of methanol and used for HPLC analysis using an InterSustainSwift C18 column (5 μm, 250 × 4.6 mm) with detection at 318 nm. Pure water and acetonitrile containing 0.04% (v/v) TFA were used as the A and B mobile phases, respectively. The gradient program used a flow rate of 1.0 mL/min at the column temperature of 40°C. Finally, the concentrations of HSAF and ATB were determined by the regression lines with *Y* = 4E-05X + 32.385 (*R*^2^ = 0.9998) for HSAF, *Y* = 6E-05X − 25.876 (*R*^2^ = 0.9991) for ATB, respectively, where X is the absorption peak area of HSAF or ATB, Y is the concentration of HSAF or ATB (mg/L).

The HSAF content in the final product was determined as follows: The eluted HSAF was concentrated under vacuum pressure, and then freeze-dried until the mass was steady. A certain amount of HSAF was dissolved in methanol to form a solution of 1,000 mg/L for the HPLC analysis, and the actual HSAF content was analyzed using the following formula:

(9) $$HSAF\left(\%\right)=C_{h}/1,000\times 100\%,$$

where *C*_*h*_ represents the HSAF concentration detected by HPLC.

### Statistical Analysis

All of the experiments were performed in triplicate, and the data are shown as mean ± standard deviation. The model parameters were calculated by OriginPro 8.6 using the linear regression method.

## Results and Discussion

### Removal of ATB From HSAF Fermentation Broth by Photodegradation

In the preliminary test, ATB in the fermentation broth was easy to degrade under natural light, while HSAF was not. This implies that ATB may be removed from the fermentation broth by photodegradation. In this study, the fermentation broth was exposed to high-intensity fluorescent light to accelerate the degradation rate of ATB in a light incubator.

**FIGURE 1:**
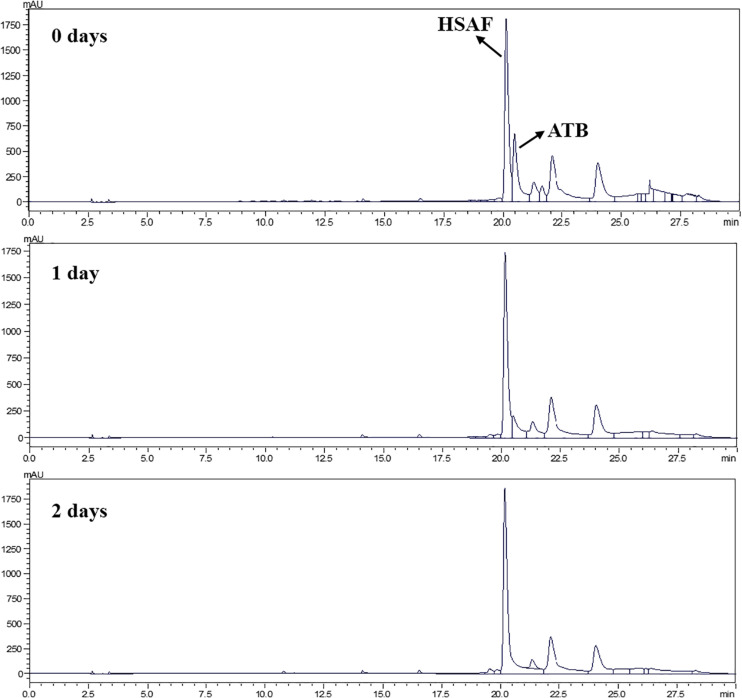
HPLC chromatogram of HSAF and ATB after illumination for 2 days.

It can be seen from the HPLC chromatogram that the ATB in the fermentation broth was found to decrease with increasing time, and no residual ATB was detected in the fermentation broth after exposure to 2 days of light (Figure 1). However, HSAF almost did not degrade under light exposure, which was consistent with the pre-experimental results. This phenomenon was mainly caused by the different ring structures between ATB (5/5-bicyclic unit) and HSAF (5/5/6-tricyclic unit). Previous studies have shown that the 5/5/6-tricyclic system is abnormally critical to the stability of the PoTeM family compounds (Huffman et al., 2010). As the main byproduct of HSAF, ATB was difficult to be separated from HSAF because of their similar structural characteristics, and this was unfavorable to the acquisition of high-purity HSAF. In this study, photodegradation proved to be an effective method to separate ATB from the fermentation broth.

Photodegradation refers to the decomposition reaction of organic molecules after the absorption of photons. This reaction has shown great advantages over the conventional ones, especially the advantage that it can be powered by sunlight, thus significantly reducing the electric power required and, therefore, the operating costs (Chatzitakis et al., 2008). Therefore, photodegradation has been widely used in various fields and for diverse applications, including the treatment of wastewater and air (Liu Y. D. et al., 2016), removal of pesticide residue in soil (Fenoll et al., 2017), and the disposal of drugs in medicine (Trawinski and Skibinski, 2017).

### Selection of Optimal MARs for HSAF Purification

Generally speaking, the adsorption and desorption capacities are critical indicators for the selection of MARs, and they are closely associated with the inherent polarities, surface areas, and average pore diameters of the adsorption resin (Yin et al., 2010). Thus, a total of 14 adsorption resins with different characteristics were selected to investigate their adsorption and desorption performances for HSAF.

**FIGURE 2:**
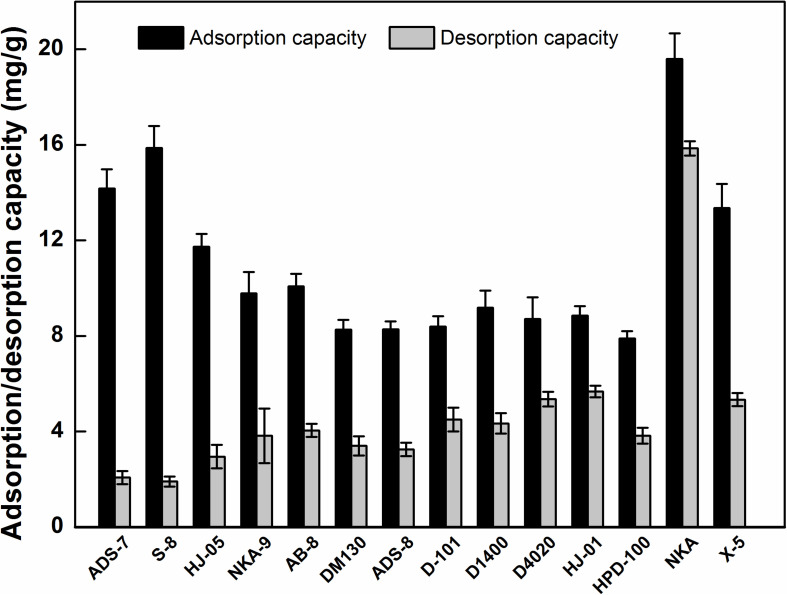
Adsorption and desorption capacities of HSAF on different MARs.

As presented in Figure 2, the strong-polar ADS-7 and S-8, medium-polar HJ-05, non-polar NKA and X5 exhibited notably higher adsorption capacity toward HSAF than the other resins; especially, the adsorption capacity of NKA reached 19.59 ± 1.08 mg/g. Considering the low polarity of HSAF, the resin adsorption result is not consistent with the principle of “like dissolves like,” which implies that the resin polarity was not a crucial factor affecting HSAF adsorption. However, these resins with high adsorption capacities had a remarkable feature: larger average pore diameters (Supplementary Table 1). A possible explanation is as follows: Two or more HSAF molecules might be bound together by intermolecular hydrogen bonds, increasing the molecular size of HSAF. The larger the average pore diameters of the resins, the easier the HSAF molecules diffuse into the MARs micropores, and the higher the adsorption capacity toward HSAF, as in the molecular sieving effect. Similar results have also been reported by other authors, who considered the matching of pore size between the adsorbent and the adsorbate to be the predominant factor affecting separation (Liu Y. F. et al., 2011). As opposed to polarity and the average pore diameter, the surface area seemed to have no effect on the adsorption of HSAF in this study. Overall, the pore diameter rather than polarity and surface area was the predominant factor influencing the adsorption of HSAF from the fermentation broth.

For the desorption process, it was observed that the higher polarity MARs resulted in a lower desorption performance. Although the strong-polar ADS-7 and S-8 featured a higher adsorption capacities toward HSAF, their desorption capacities were rather low (2.07 ± 0.17, 1.91 ± 0.21 mg/g), resulting in low desorption ratios (14.61 ± 1.19 and 12.01 ± 1.32%, respectively). This is probably due to the strong polarity and adsorption ability, which made it difficult to elute HSAF from the polar MARs, compared with the elution using other resins.

Considering the adsorption and desorption performances of MARs, the non-polar NKA was selected as a suitable resin for the separation of HSAF from the fermentation broth. At present, there are few reports on separating active compounds using the NKA resin, and NKA is usually utilized as a support for lipase immobilization to enhance enzyme activity and catalytic performance (Liu T. et al., 2011; Li et al., 2013). In the current study, NKA showed a strong adsorption capacity for HSAF in the fermentation broth, which may increase the application range of NKA resin.

### Static Adsorption and Desorption Kinetics of HSAF on NKA Resin

**FIGURE 3:**
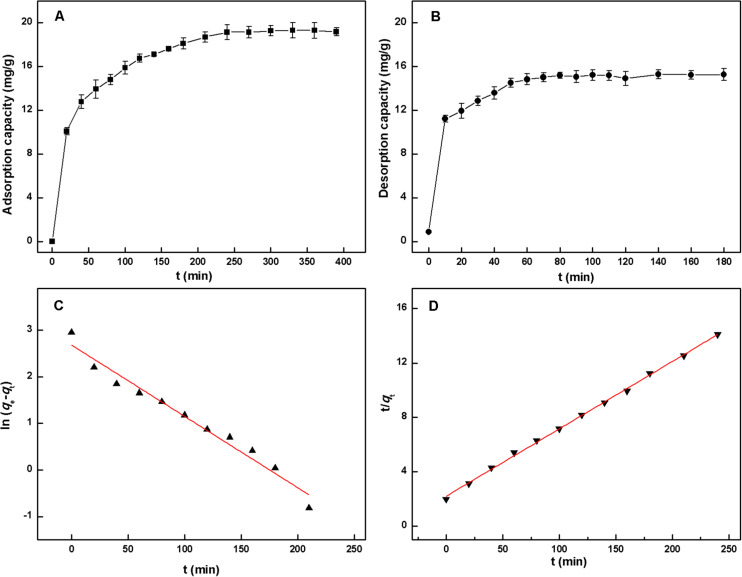
Static adsorption kinetic **(A)** and desorption kinetic **(B)** curves of HSAF on the NKA; linear correlation plots based on pseudo-first-order kinetic **(C)** and pseudo-second-order kinetic model **(D)**.

Adsorption kinetics describes the adsorption rate of a solute on an adsorbent and governs the contact time of the sorption reaction. It has proved to be an important characteristic to evaluate the efficiency and feasibility of MARs on practical applications (Liu et al., 2010). Therefore, the adsorption kinetics of HSAF on NKA resin was investigated, and the result is shown in Figure 3A. It was evident that the adsorption capacity of HSAF on NKA resin increased with the extension of adsorption time until equilibrium. In the first 20 min, the adsorption capacity of NKA exhibited a linear and instantaneous increasing trend due to the high diffusivity of solute molecules into the resin micropores. Then the adsorption increased slowly and reached equilibrium at approximately 240 min, indicating that the adsorption of HSAF onto NKA resin belongs to a slow process. This result might be caused by the following two reasons:(1) the high intra-particle mass transfer resistance within the resins following the increase in the amount of adsorbed adsorbate and (2) too many impurities and low concentration of HSAF in the fermentation broth.

To better illustrate the adsorption mechanism, the pseudo-first-order model and pseudo-second-order model were employed to describe the adsorption process. By plotting ln (*q*_*e* –_ *q*_t_) against t for the pseudo-first-order equation and t/*q*_t_ against t for the pseudo-second-order equation, two straight lines and model rate constants were obtained, as shown in Figures 3C,D and Supplementary Table 2. Considering the obtained correlation coefficients *R*^2^, the pseudo-second-order kinetics model (0.9987) was more suitable for describing the adsorption behavior of HSAF onto the NKA resin than the pseudo-first-order model (0.9680). This result indicates that the concentrations of HSAF and NKA resin were both involved in the adsorption rate according to the principle of the pseudo-second-order model, indicating that the adsorption mechanism was probably chemiadsorption. Moreover, the calculated *q*_*e*_ (20.19 mg/g) was very close to the experimental value, implying that the adsorption process of HSAF on NKA resin conformed to the pseudo-second-order kinetic model.

The desorption kinetics of HSAF on NKA resin was also carried out, as shown in Figure 3B. The desorption was completed within 60 min (14.82 ± 0.51 mg/g), suggesting that the desorption process of HSAF from NKA was very quick and effective when ethanol was used as eluent. Ethanol has been regarded as a preferable desorbent for MARs and is widely utilized, due to its many advantages, such as low price, non-toxicity to the human body, and recyclability (Wu et al., 2015).

### Adsorption Isotherms of HSAF on NKA Resin

**FIGURE 4:**
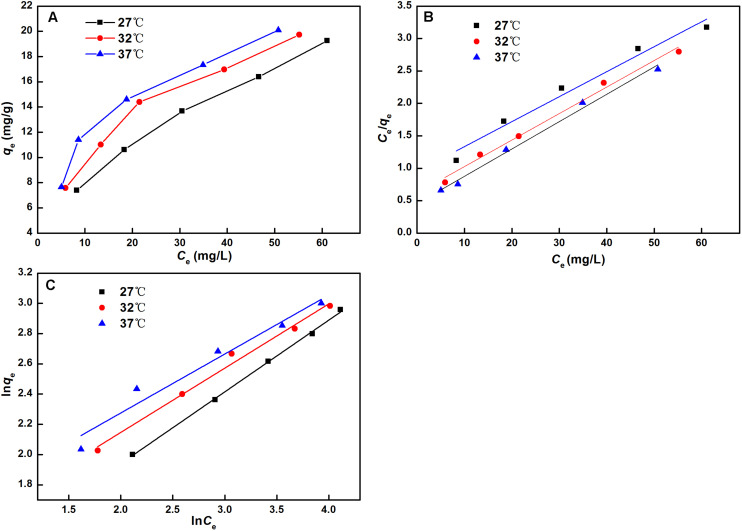
Adsorption isotherms **(A)**, linear correlations based on the Langmuir **(B)** and Freundlich **(C)** models of HSAF on the NKA at different temperatures.

To better illustrate the adsorption properties of NKA, equilibrium adsorption isotherms of HSAF on NKA resin were investigated at three different temperatures, and the results are shown in Figure 4A. With the increase in the initial concentration, the adsorption capacity of HSAF first increased rapidly, and then the trend gradually slowed down and reached a limit. In addition, the adsorption capacity of HSAF onto NKA increased with the temperature increasing from 27 to 37°C at the same sample initial concentration, implying that the adsorption was an endothermic process. A possible explanation is that the higher temperature could accelerate the mobility of molecules, thus enhancing the adsorption performance. Positive effects of temperature have also been reported in previous studies (Liu T. et al., 2011; Kim et al., 2014). Considering these results, an initial HSAF concentration of 300 mg/L and a temperature of 37°C were selected for further research. Under these conditions, the maximum adsorption capacity and ratio for HSAF were 20.10 ± 1.20 mg/g and 83.07 ± 4.96%, respectively.

Furthermore, the Langmuir and Freundlich models were adopted to fit the experimental data (*qe*, *Ce*) obtained under different temperatures and describe how HSAF interacted with the NKA resin. The linearity of each model is presented in Figures 4B,C, and the model parameters obtained from the regression equations at different temperatures are summarized in Supplementary Table 3. Within the measured temperature ranges, the *q*_*m*_ values and equilibrium constant *K*_*L*_ in the Langmuir equation increased with the increment of temperature, implying that elevated temperature was beneficial for the thermal motion of HSAF and adsorption onto the NKA resin, which was consistent with the experimental results. The value of n in the Freundlich equation was the measure of the adsorption driving force and energy distribution of sorption sites. Generally speaking, the adsorption is favorable when 0 < 1/*n* < 1 but not when 1/*n* ≥ 1. In the present work, the values of 1/n were all between 0 and 1 at different temperatures (Supplementary Table 3), indicating that HSAF in the fermentation broth can easily be adsorbed on the NKA resin. In terms of the linear regression correlative coefficient (*R*^2^), the values of the Langmuir model were all higher than those of the Freundlich model at the same temperature, suggesting that the Langmuir isotherm could reasonably describe the adsorption process. The Langmuir isotherm is widely known and the most frequently used for the adsorption of solutes from a solution due to its simplicity. It assumes a homogeneous distribution among the adsorption sites at different energies and the absence of mutual interaction between adsorbed molecules (Yin et al., 2010). Hence, the adsorption process of HSAF only occurred in the local monolayer coverage of the NKA resin.

### Dynamic Adsorption and Desorption of HSAF on NKA Resin Column

#### Dynamic Adsorption Breakthrough Curve

In the process of dynamic adsorption, once the adsorption process reaches the breakpoint, the adsorption capacity decreases rapidly, even disappears, resulting in the solutes leaking from the resin (Fang et al., 2019). Hence, in this study, it was essential to construct the dynamic breakthrough curve to calculate the suitable flow rate and the loading volume of the sample solution.

**FIGURE 5:**
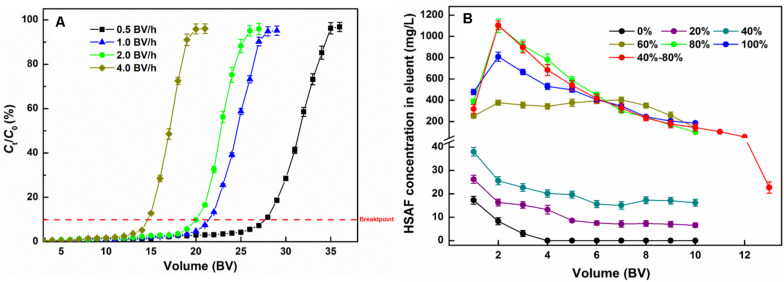
Dynamic adsorption breakthrough curve **(A)** and desorption curve **(B)** of HSAF on the NKA packed column.

As displayed in Figure 5A, the breakthrough curves at different flow rates show similar tendencies; the HSAF concentration in the effluent slowly increased at the beginning of the adsorption, then rapidly increased beyond the breakpoint until it plateaued. In our study, the breakpoint was defined as the 10% ratio of the exit solute concentration to the inlet solute concentration (*C*_t_/*C*_0_). At the 10% breakpoint, the breakthrough volumes of HSAF on the NKA resin column were 28, 22, 20, and 15 BV at flow rates of 0.5, 1.0, 2.0, and 4.0 BV/h, respectively, and the corresponding adsorption capacities were calculated as 34.72 ± 1.85, 26.57 ± 1.46, 25.12 ± 1.32, and 18.23 ± 1.13 mg/g. The result showed that increasing the flow rate had a negative effect on dynamic adsorption capacity of HSAF on NKA resin, because adsorbate molecules had no sufficient time to interact with active sites at the surface of resins and vice versa. Thus, as the flow rate increased, the contact time between HSAF molecules and NKA resin became shorter, leading to inadequate adsorption. Similar results have also been observed in other studies (Wei et al., 2011; Chang et al., 2012). However, the breakpoint was also delayed with a lower flow rate, which means the loading process was prolonged. When the flow rate increased from 1.0 to 2.0 BV/h, the adsorption capacities only slightly declined. However, the loading time could be reduced by half, thus greatly increasing the adsorption efficiency.

Considering the adsorption capacity and time consumption, 2.0 BV/h was selected as the appropriate sample flow rate. Under this condition, the loading volume of the HSAF fermentation broth on the NKA column was determined to be 20 BV (144 mL), and chosen for further experiments.

#### Dynamic Desorption Curve

To decrease the consumption of reagents and make desorption more efficient, the influences of flow rate, the concentration of aqueous ethanol solution, and volume on the desorption process were investigated. Since the effect of flow rate on the desorption process was similar to that on the adsorption process, the flow rate was determined as 2.0 BV (the detailed results are not shown).

**FIGURE 6:**
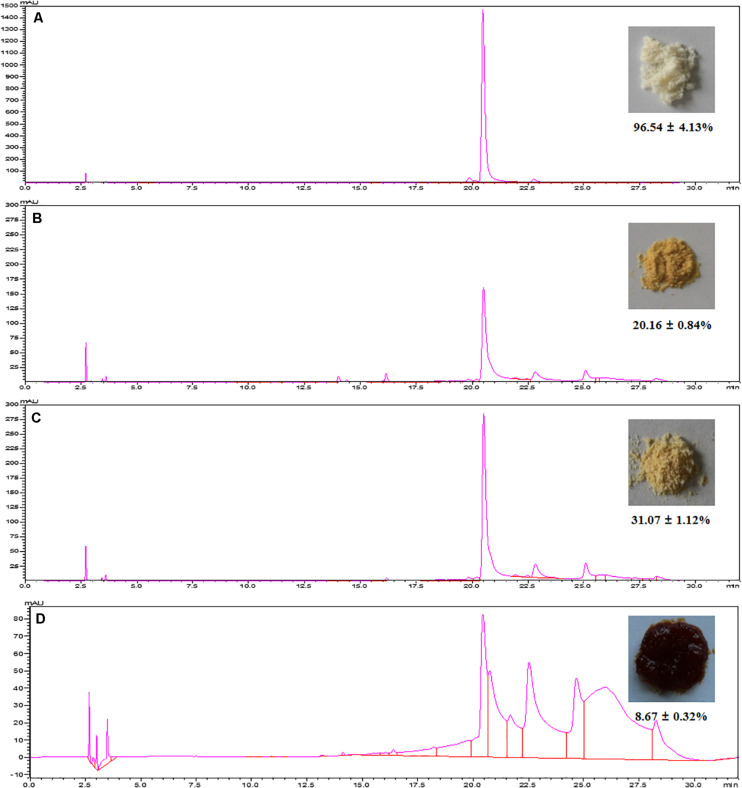
Chromatograms and the pictures of the final HSAF products with different methods. **(A)** The preparative HPLC; **(B)** the NKA adsorption with 80% ethanol solution elution; **(C)** the NKA adsorption with stepwise elution; **(D)** ethyl acetate extraction.

As shown in Figure 5B, HSAF could not easily be eluted by water and low concentrations of aqueous ethanol. When the ethanol concentration was over 40%, the HSAF concentration in the eluent increased sharply; it reached a peak value at 80% aqueous ethanol and then decreased at 100% ethanol. Since at 40% aqueous ethanol, little HSAF was desorbed, this concentration of aqueous ethanol could be used to remove impurities and high-polarity compounds from the resin before elution. Therefore, for the desorption process, 40 and 80% aqueous ethanol are selected as the concentrations of the cleaning solution and desorption solution, respectively. Therefore, a stepwise elution procedure is established and the dynamic desorption curve is also displayed in Figure 5B. When the NKA resin was flushed with 40% aqueous ethanol, there were some polar compounds (peak group with retention times between 13 and 16 min at HPLC) in the effluent (Figure 6B), and their amounts gradually decreased with the increase in the amount of reagent used. Finally, these compounds were completely removed with the consumption of 4 BV 40% aqueous ethanol. Then 80% aqueous ethanol was used to elute HSAF from the NKA. The HSAF concentration in the eluent increased rapidly to the maximum at 2 BV and decreased to a quite low level at 13 BV (22.73 ± 2.44 mg/L). Thus, the elution volume was determined to be 12 BV (86.4 mL). Under this condition, the desorption capacity and desorption ratio of NKA resin toward HSAF reached 23.28 ± 1.44 mg/g and 92.66 ± 5.95%, respectively. Compared with the single-pass elution (80% ethanol solution), the stepwise elution could acquire lighter appearance and higher content of HSAF (Figures 6B,C).

In summary, the optimal conditions for the elution scheme were 4 BV 40% and 12 BV 80% aqueous ethanol at a flow rate of 2.0 BV/h.

#### Comparison of HSAF Contents Obtained Using Resin Adsorption and Traditional Methods

To validate the efficiency of MARs, the chromatogram, appearance, and content of the final HSAF product were compared with those obtained using the conventional methods. As shown in Figure 6, the preparative HPLC yielded 96.54 ± 4.13% purity of HSAF, which appeared as milky white power (Tang et al., 2018a). However, this chromatographic separation method can only be used in small-scale production because of its high cost and time consumption. The extraction method by ethyl acetate yielded a brown paste product with a low content of 8.67 ± 0.32%. Purification using the NKA resin yielded a light yellow final HSAF product, which was closer to the color of pure HSAF.

In addition, the HSAF content reached 31.07 ± 1.12%, a 3.58-fold increase compared with that obtained by ethyl acetate. These results indicate that the MARs adsorption prevented the interference of various pigments in the process of organic solvent extraction, which could improve the HSAF purity. Although the purity is not yet at a high level, it has meet the initial requirements of agricultural application.

## Conclusion

In this study, ATB was first removed from the HSAF by photodegradation. Then NKA resin was selected as the most suitable resin for the adsorption of HSAF from the fermentation broth, and the static and dynamic adsorption characteristics were systematically investigated to obtain the optimal separation parameters. After purification, the content of HSAF in the final product (31.07 ± 1.12%) was 3.58-fold higher than that obtained by the conventional chemical method (8.67 ± 0.32%). Therefore, this method via photodegradation and NKA resin adsorption is an effective process for the separation of HSAF.

## Data Availability Statement

The original contributions presented in the study are included in the article/Supplementary Material, further inquiries can be directed to the corresponding author/s.

## Author Contributions

BT, LW, and FL conceived and designed the experiments. BT, WS, and JW performed the experiments. BT and LW analyzed the data and wrote the manuscript. YZ and FL reviewed and edited the manuscript. All authors contributed to the article and approved the submitted version.

## Conflict of Interest

The authors declare that the research was conducted in the absence of any commercial or financial relationships that could be construed as a potential conflict of interest.
