# Supplementary material for: Separation of Heat-Stable Antifungal Factor From Lysobacter enzymogenes Fermentation Broth via Photodegradation and Macroporous Resin Adsorption
Source: Front Microbiol. 2021 May 13;12:663065. doi: 10.3389/fmicb.2021.663065 (PMC8155363; doi:10.3389/fmicb.2021.663065)
Supplement: Supplementary file 1 [file Table_1.DOCX]

Supplementary Material

## Supplementary Tables

**Table S1** Physicochemical properties of the tested MARs

| **Type** | **Polarity** | **Surface area (m^2^/g)** | **Average pore diameter (nm)** |
| --- | --- | --- | --- |
| ADS-7 | Strong-polar | ≥100 | 25-30 |
| S-8 | Strong-polar | 320-420 | 28-30 |
| HJ-05 | Medium-polar | ≥880 | 12-15 |
| NKA-9 | Polar | 170-250 | 15.5-16.5 |
| AB-8 | Weak-polar | 480-520 | 13-14 |
| DM130 | Weak-polar | 500-550 | 9-10 |
| ADS-8 | Non-polar | ≥450 | 12-16 |
| D-101 | Non-polar | 550-600 | 9-11 |
| D1400 | Non-polar | 900-1100 | 8.4-9.4 |
| D4020 | Non-polar | 540-580 | 10-10.5 |
| HJ-01 | Non-polar | ≥780 | 12-14 |
| HPD-100 | Non-polar | ≥650 | 8.5-9 |
| NKA | Non-polar | 570-590 | 20-22 |
| X-5 | Non-polar | 500-600 | 29-30 |

**Table S2** Adsorption kinetic parameters of pseudo-first- and second-order model

| **Models** | **Equations** | **Parameters** | | |
| --- | --- | --- | --- | --- |
|  |  | ***q*_e_ (mg/g)** | ***K*** | **R^2^** |
| Pseudo-first-order model | ln (*q*_e_- *q*_t_) =-0.0153t+2.68151 | 14.61 | 1.53×10^-2^ | 0.9680 |
| Pseudo-second-order model | t/ *q*_t_ =0.04954t+2.2113 | 20.19 | 1.84×10^2^ | 0.9987 |

**Table S3** Adsorption isotherm equation and parameters of HSAF on NKA resin

| **Temperature** | | **27°C** | **32°C** | **37°C** |
| --- | --- | --- | --- | --- |
| Langmuir equation | Linear equation | *C*_e_/*q*_e_=0.03852*C*_e_+ 0.94812 | *C*_e_/*q*_e_=0.04086*C*_e_+ 0.61367 | *C*_e_/*q*_e_=0.04216*C*_e_+ 0.45161 |
|  | *q*_m_ | 25.9605 | 24.4738 | 23.7192 |
|  | *K_L_* | 0.04063 | 0.06658 | 0.09335 |
|  | R^2^ | 0.96837 | 0.98877 | 0.99106 |
| Freundlich equation | Linear equation | ln*q*_e_= 0.47428ln*C*_e_+ 0.99179 | ln*q*_e_=0.42484ln*C*_e_+ 1.29682 | ln*q*_e_=0.38976ln*C*_e_+ 1.49541 |
|  | *K_F_* | 2.696056 | 3.657647 | 4.461165 |
|  | 1/n | 0.47428 | 0.42484 | 0.38976 |
|  | R^2^ | 0.99848 | 0.98573 | 0.95119 |
